# Delay in hospital discharge of trauma patients in a University Hospital in Egypt: A prospective observational study

**DOI:** 10.1016/j.afjem.2021.06.008

**Published:** 2021-10-28

**Authors:** Islam El-Abbassy, Wafaa Mohamed, Hazem Mohamed El-Hariri, Maged El-Setouhy, Jon Mark Hirshon, Mohamed El-Shinawi

**Affiliations:** a General Surgery Department, Faculty of Medicine, Ain Shams University, Abbassia, Cairo, Egypt; b Raigmore Hospital, NHS Highland, Old Perth Road, Inverness, UK; c Institute of Medical Sciences, University of Aberdeen, King's College, Aberdeen, UK; d Department of Community, Environmental and Occupational Medicine, Faculty of Medicine, Ain Shams University, Abbassia, Cairo, Egypt; e Community Medicine Department, National Research Centre, Ad Doqi, Dokki, Cairo, Egypt; f Department of Family and Community Medicine, Faculty of Medicine, Jazan University, Jazan, Saudi Arabia; g Department of Emergency Medicine, University of Maryland School of Medicine, Baltimore, MD, United States; h Galala University for International Cooperation Community Services and Environmental Department, Galala University, Sokhna, Suez Governorate, The city of Galala Plateau, Governorate of Suez on the coast of the Red sea El Galala Maritime Plateau, Egypt

**Keywords:** Delayed discharge, System-related factors, Family-related factors, Medical factors, Time to discharge

## Abstract

**Introduction:**

“Delayed discharge” is defined as patients who remain hospitalised beyond the time of being fit for discharge after a decision of discharge has been made by the managing team. There is no standardised amount of time defining delayed discharge documented in the literature, and there is a lack of evidence about this topic in Egypt. This study is a quality improvement project aiming to identify the factors associated with discharge delays at a single centre in Egypt in order to address this issue.

**Methods:**

A prospective observational study included all trauma patients admitted to a University Hospital in Egypt over two months. The time of the decision of discharge and actual discharge time were recorded by reviewing patients' medical records. The patients and their caregivers were asked to fill in a questionnaire about the reasons for delayed discharge. Potential reasons for the delayed discharge were classified into system-related, medical and family-related factors.

**Results:**

The study included 498 patients with a median age of 41 years (9–72). The median time from discharge decision until actual discharge was 3 h. System-related factors were documented in 48.8% of cases, followed by medical factors (36.3%), and family-related factors (28.1%). When controlling for age, gender and injury severity score using a logistic regression analysis, longer time to discharge (≥3 h) showed a stronger association with medical factors [adjusted OR (95% CI) = 5.44 (2.73–10.85)] and family-related factors [adjusted OR (95% CI) = 7.94 (3.40–18.54)] compared to system-related factors [adjusted OR (95% CI) = 2.20 (1.12–4.29)].

**Discussion:**

Although system-related factors were more prevalent, medical and family-related factors appear to be associated with longer discharge delays compared to system-related factors.

## African relevance

● There are limited resources in Africa to provide free medical services to the public, and Egypt is no exception.
● Inappropriate bed use and prolonged stay increase the financial burden on the health services where hospital admissions account for a significant amount of total healthcare costs.
● Improvements in acute care bed management could result in significant savings and being able to define delayed discharge will help start a quality control process to address discharge delays and their potential causes.

## Introduction

A “delayed discharge” is defined as a hospital inpatient who continues to occupy a bed beyond the time of being judged clinically fit for discharge after a decision of discharge has been made by the managing team. The discharge decision is usually made as part of a multi-disciplinary process in which some patients might need a review by other specialities before discharge [1]. Other terms used to describe delayed discharge include ‘inappropriate acute bed use’ and ‘bed blocker’ of which the latter term is most frequently used [2]. Despite being a fairly common problem in healthcare settings; to our knowledge, there is no standardised definition of what constitutes delayed discharge in the literature.

Delays in discharge have raised numerous concerns over the past few years [3]. These include an increased risk of infection, a reduced quality of life particularly for elderly patients, and a potential waste of economic and human resources [4]. Delayed discharge is associated with adverse effects on both the patients and the health care staff. At the patient level, there may be an increased risk of falls, hospital-acquired infections and mental health problems [5]. As for the health care staff, this may be associated with stress and diversion from a primary focus on patient care [6].

The percentage of occupying the hospital beds by patients after being fit to discharge (delayed discharge) was reported to range between 15 and 50% in the Netherlands [5] and 8.5% in Scotland [7]. The National Health Service (NHS) of England experiences approximately £100 m per year in costs associated with delayed discharge [8]. The increased cost of delayed discharged was reported by different studies and was attributed to occupying beds, needing to pay for nursing staff and other administrative costs 9., [10]. In a study done by Thomas S et al. in the USA, the total cost of delayed discharge per year was reported to be about $715,403 [10].

University hospitals in Egypt have a limited budget and they often provide free services to the public 11., [12]. Inappropriate bed use and prolonged stay increase the financial burden on the health services especially in Egypt, where hospital admissions account for a significant amount of total healthcare costs [12]. Therefore, improvements in acute care bed management could result in significant savings.

Several studies have identified factors associated with delayed discharge and classified them into medical, familial and system-related 9., [13], 14.. Delayed discharge is described as a multi-factorial problem that requires effective teamwork within the hospital and coordination between health care providers, caregivers at home and social care services [6].

Studies conducted in different countries, such as the USA, the UK and Canada, have found that delays in discharge are often related to system-related delays due to difficulties in transferring patients to rehabilitation facilities or back home 9., [10], 15., [16]. Although some studies discussed the issue of delayed discharge in western countries, this issue has not been well discussed in African ones. It is to be noted that delays associated with arrangements for referring patients to rehabilitation facilities were not considered in our study since the availability of such facilities is limited in Egypt.

To address the issue of not having a standardised definition of delayed discharge in the literature, a clinical administrative panel (including four senior general surgery and orthopaedic consultants, along with two senior nurses), agreed that “three hours” was the maximum acceptable time until actual patient discharge once a discharge order has been made. By reviewing the existing literature and confirming the non-availability of a standardised definition for delayed discharge, in addition to monitoring and assessing the flow of work required to finish the discharge process of patients in our hospital, the panel felt that 3 h is a reasonable time to cover any final medical procedures and paperwork required before actual discharge. This study aimed to document the amount of time taken for trauma patients to leave the hospital once the order to discharge had been made by the attending physician and to determine the factors associated with discharge delays. This could help provide evidence to the expert panel concerning the three-hour cut off value set to define delayed discharge; and will also help start a quality improvement project to address discharge delays and their potential causes.

## Methods

This study was conducted at Ain Shams University Surgical Hospital which is one of the major tertiary educational centres in Egypt. It has a total capacity of 520 beds and it offers free service to the public. It constantly receives new admissions as well as referrals from other health facilities. According to the hospital records, the occupancy rate ranges between 90% and 110%. Trauma cases are usually occupying around 40% of the total hospital capacity.

We limited our study to trauma cases over a period of two months, to allow for a focused analysis of the causes of delayed discharge in this busy hospital.

A prospective observational study conducted during two separate months (August 2016 and January 2017). These two months were randomly selected from the two main seasons in Egypt [summer (May to October) and winter (November to April), respectively]. The two seasons were represented in case there were differences in the pattern of admissions regarding age, gender, or Injury Severity Score (ISS).

All admitted trauma patients (of all ages and both sexes) during the specified study duration were included. Patients were followed from admission until discharge. Deceased patients were excluded from the study. All included patients were discharged from the hospital wards to their homes.

The possible reasons for delayed discharge in our hospital were discussed by the hospital administrative panel who developed an interview questionnaire used in our study.

The developed questionnaire about the potential reasons for delayed discharge was composed of twelve questions which were answered by the patients and their caregivers. Reasons for delayed discharge were classified according to the previous literature into system-related, medical, and family-related factors 9., [13], 14.. System-related factors included: delayed paperwork, delayed consultation by other specialities before discharge, delayed issuing the discharge order, and delays caused by nursing. Medical factors included: delayed wound dressing, delayed drainage-tube removal, treatment of co-morbidities, stoma care and daily wound dressing for those who have complicated wounds and cannot manage to dress their wounds by themselves at home. Family-related factors included: delayed pick up from the hospital by relatives, living alone with single care, and living in remote areas.

A data extraction sheet was used to collect data from the patients' medical records after discharge. Recorded data included age, sex, date of admission, date of discharge, length of hospital stay (LOS), whether surgery occurred, and whether the patient was admitted to the Intensive Care Unit (ICU). Data about the nature of the injury were collected to calculate the Injury Severity Score (ISS). Data about co-morbidities were collected to calculate the Charlson Co-morbidity Index (CCI) [17], [18]. Additionally, the time of the discharge decision documented in the patients' notes during the ward round (which is the starting point to calculate the delay time), the time the discharge order was issued, and the actual discharge time were all recorded. Any clinical consequences for delayed discharge were reported, such as hospital-acquired infections, falls or mental health problems. Data collection tools were reviewed by the clinical administrative panel for face validity.

Statistical analysis was performed using SPSS version 23 (IBM Corp, 2016. IBM SPSS statistics: version 23.0). Qualitative variables were presented in the form of frequencies and percentages. Ordinal variables were presented as medians with inter-quartile range (IQR). The Mann Whitney *U* test and Spearman correlation were used for univariate analysis of factors associated with delayed discharge. Binary multiple logistic regression analysis was used to examine the role of the three categories: system-related, medical, and family-related factors in prolonging time to discharge. Variables with P-values ≤0.05 were introduced simultaneously in the model. The final model was obtained by removing variables with the highest P-values one by one and using the Akaike information criterion (AIC) to select the model that fits the data best. The model with the lowest AIC value was selected.

Under the 1964 Helsinki declaration and its later amendments, the study protocol was approved by: The Institutional Review Board (IRB) of Ain Shams University, Cairo, Egypt. Date: 23/11/2014. Reference: IRB 00006379; and the Institutional Review Board (IRB) of the University of Maryland, Baltimore, USA. Date: 04/02/2015. Reference: HP-00062968.

Written informed consent was obtained from all participants included in the study.

## Results

A total of 498 patients, 240 patients in summer and 258 in winter, were included in the study. There were no statistically significant differences between the patients admitted in August and those admitted in January regarding age, gender or ISS. The patients' median age was 41 years (IQR, 28–50) with a range of 9–72 years. Most patients 306 (61.4%) were males. Sixty percent of the patients underwent surgery, and 211 (42.4%) were admitted to the ICU. The median ISS was twelve (IQR, 1–20) and the median CCI score was one (IQR, 0–2) (Table 1, Table 2).

**Table 1:** Demographic characters of patients included in the study.

		Number	Percentage
Gender	Male	306	61.4%
	Female	192	38.6%
OR admission	Yes	299	60.0%
	No	199	40.0%
ICU admission	Yes	211	42.4%
	No	287	57.6%
Season	Summer	240	48.2%
	Winter	258	51.8%
Discharge planning	Formal^a^	396	79.5%
	Interdisciplinary^b^	102	20.5%
	**Total**	**498**	**100%**

OR: operating room; ICU: Intensive Care Unit.

a Formal: surgical discharge without the need for input from other specialities.

b Interdisciplinary: input from other specialties was required.

**Table 2:** Characteristics of the study population (N = 498).

	Median	IQR	Range
Age (years)	41	28–50	9–72
ISS	12	1–20	1–50
CCI	1	0–2	0–6
LOS (days)	4	1–8	1–28
Time to discharge (in hours)	3	2–6	0.25–336

IQR: inter-quartile range; ISS: Injury Severity SCORE; CCI: Charlson Co-morbidity Index; LOS: length of stay.

The median LOS was four days (IQR, one to eight days), and the median time to discharge after the decision of discharge was made was 3 h (IQR, 2 to 6 h). The discharge of nearly half of the patients (238 (47.79%)) was delayed for 3 to 10 h after a discharge order has been made, and 89 (17.87%) were delayed for 24 h or more. System-related factors were reported in 243 (48.8%) patients, followed by medical factors that were reported in 181 (36.3%) patients. Family-related factors were reported in only 140 (28.1%) patients.

Univariate analysis showed that the median time to discharge was longer for females, patients who underwent surgery, patients admitted to the ICU, and patients with interdisciplinary discharge planning (Table 3).

**Table 3:** Median time to discharge (in hours) described by demographic characters and type of care (N = 498).

		Time to discharge		P^a^ value
		Median	IQR	
Gender	Male	3	1–5	<0.001
	Female	5.5	3–24	
OR admission	No	2	1–3	<0.001
	Yes	6	3–24	
ICU admission	No	2	1–3	<0.001
	Yes	7	4–24	
Season	Summer	3	2–6	0.991
	Winter	3	2–7	
Discharge planning	Formal	3	2–5	<0.001
	Interdisciplinary	24	5–48	

a Mann Whitney *U* test.

The median time to discharge was significantly longer for patients with delays related to the treatment of co-morbidities, stoma care, and daily wound dressing. All family-related factors were significantly associated with a longer median time to discharge. Among system-related factors, delayed consultation was the one associated with an increased median time to discharge (Table 4).

**Table 4:** Median time to discharge (in hours) by reasons for the delay.

Delay reason		Yes		No		P⁎ value
		Median	IQR	Median	IQR	
Medical	Delayed wound dressing	4	3–6	3	2–7	0.054
	Delayed tube removal	4	2–6	3	2–7	0.688
	Treatment of co-morbidities	24	7–72	3	2–6	<0.001
	Stoma care	48	48–48	3	2–6	<0.001
	Daily wound dressing	72	48–72	3	2–6	<0.001
Family	Delayed pick up	6	4–10	3	2–6	<0.001
	Living alone	48	48–72	3	2–6	<0.001
	Living in remote areas	17	6–24	3	2–5	<0.001
System	Delayed paperwork	1	0.5–3	4	2–7	<0.001
	Delayed consultation	6.5	5–24	3	2–6	<0.001
	Delayed written discharge order	2	2–2	4	2–7	<0.001
	Delays by nursing	3	2–6	4	2–7	0.374
Any medical related delay		6	4–48	3	1–5	<0.001
Any familial related delay		6	5–24	3	1–5	<0.001
Any system-related delay		3	1–4	5	3–24	<0.001

⁎ Mann Whitney U test, P ≤ 0.05 considered significant.

There was a weak correlation between age (ρ = 0.34 P < 0.001), CCI (ρ = 0.38, P < 0.001), and time to discharge. Time to discharge was moderately correlated with ISS (ρ = 0.61, P < 0.001), and LOS (ρ = 0.69, P < 0.001).

A binary multiple logistic regression analysis showed that age had no significant effect on delayed discharge for more than 3 h. The factors that had a significant effect on delayed discharge more than 3 h according to the strength of relation from strongest to weakest were familial delays (β = 2.072), medical delays (β = 1.694), ISS >15 (β = 1.556), female gender (β = 1.042) and system delays (β = 0.787) (Table 5).

**Table 5:** Logistic regression analysis for predictors of delayed discharge (≥3 h) following a discharge order.

	B	P value	OR	95% CI for OR	
				Lower	Upper
Age 18–59^a^	0.616	0.287	1.85	0.60	5.76
Age ≥ 60^a^	0.115	0.875	1.12	0.27	4.67
Female	1.042	<0.001	2.84	1.70	4.74
ISS > 15	1.556	<0.001	4.74	2.49	9.02
Medical delays	1.694	<0.001	5.44	2.73	10.85
Familial delays	2.072	<0.001	7.94	3.40	18.54
System delays	0.787	0.021	2.20	1.12	4.29

The dependent variable, delayed discharge ≥3 h.

a Reference category is age < 18 years.

No clinical consequences were reported for the patients who experienced delayed discharge.

## Discussion

There is no standard definition for delayed hospital discharge in the literature, however, some studies attempted to define delayed discharge in different ways. One study in the USA used insurance Diagnosis Related Group–based time points [9], another study used a 24-hour cut off point to define delayed discharge [19]. Both studies acknowledged that discharge delay duration varies between practices and institutes.

In the current study, we calculated delays from the time of the decision of discharge until the patient left the hospital, which had a median of 3 h. This corresponded to the definition of acceptable delay set by the clinical administrative panel in our hospital.

In our study, system-related factors were reported in nearly half of the patients. This is relatively higher than a study in the USA has found; where only a quarter of patients experienced system-related delays [9].

Factors related to rehabilitation facility arrangements post-discharge (system-related factors) were found to be strongly associated with discharge delays in the USA and the UK 5., 9., 15., 19.. Such facilities are not as common in Egypt. Excluding these factors from our study, makes other system-related factors in our hospital more prominent than those in the other studies. This indicates that there are more delays in our hospital related to delayed paperwork, delayed other specialities consultation, delayed written discharge orders and delays caused by nursing.

The equivalent of delays related to post-discharge rehabilitation facility arrangements in the current study was family-related arrangements in terms of delayed pick up from the hospital by relatives, living alone with single care, and living in remote areas requiring a longer time to arrange for a proper transportation method. Family-related factors were reported by only a quarter of the patients in the current study; however, they were significantly associated with longer discharge delays compared to other factors. Despite being the least reported category in this study, family-related factors were five times more frequent than reported by the NHS in Scotland [5]. Compared to developed countries, where rehabilitation facilities are more widely available, post-discharge care is less institutionalised in developing countries. Since a lot of responsibility for post-discharge patient care is transferred to family members, hospital staff needs to communicate more closely with patients' families to help them be more readily prepared to provide post-discharge care.

Medical-related factors were reported in over one-third of the patients in the current study. Studies in the USA and the UK have similarly emphasised the role of medical-related delays to discharge 7., 9..

Payer related issues and insurance provider delays were among the main reasons for delayed discharge in the USA 9., 19.. But in the current study, the hospital offered free service to the patients, and hence, payment related issues were not encountered; although the situation might be different in other settings such as in private hospitals.

In our study, age only correlated weakly with delayed discharge, whereas, in other studies, age was one of the factors that influenced delayed discharge [10], 15.. Our study was restricted to trauma patients who were relatively younger compared to mixed cases in other studies that included non-trauma patients as well as patients admitted for various medical indications of older age groups.

We found in our study that the time to discharge for trauma patients was significantly longer for trauma patients with an ISS > 15. Other studies in the USA and Iran also showed similar results [10], 20.. In contrast, Hwabejire et al. in their retrospective study of 3237 trauma patients found that ISS was not the main factor in delaying hospital discharge [9].

In our study, it was found that female patients had a longer median time to discharge. A similar finding was also reported by Bai et al. [15].

No clinical consequences were reported for the patients who experienced delayed discharge in our study; however, delayed discharge was negatively reflected on the rate of turnover of the acute beds in our busy tertiary hospital that provides free healthcare services for thousands of patients every year. Therefore, delayed patients' discharge is a pressing issue that needs to be addressed to improve patient care and to avoid any excess costs.

Accordingly, we need to find appropriate solutions for various reasons of delayed discharge and a quality control process should be put in place to investigate all these reasons to be able to come up with the proper solutions.

From the given reasons for delays, some solutions were suggested that need to be explored. The suggested solutions include having junior doctors prepare discharge paperwork in advance for patients who are expected to be discharged, providing dedicated unit secretaries to appropriately care for the discharge paperwork, and increasing the nursing staff on the unit to finish all the required dressings and other pending medical issues that may delay discharge.

Although family-related factors were the least commonly reported reasons for the delay, they were strongly associated with delays in discharge beyond 3 h. One possible intervention to reduce family-related delays might be by providing earlier notice of discharge to patients and their families. There is also a need to facilitate rapid, proper, and safe transport of patients to their homes. Moreover, nursing homes and rehabilitation facilities should be encouraged by the government to help elderly patients, particularly those who live alone, so that they can find a safe environment to live (at least temporarily) following discharge.

One of the limitations of our study was that it included only trauma patients in one centre. To fully understand the extent and pattern of hospital discharge delays in Egypt, more inclusive studies in other healthcare settings and other specialities are needed. Also, this quality improvement project may inspire other hospitals in Egypt to conduct similar quality improvement projects to identify delays at their facilities. Another limitation to the study was that it was restricted to a two-month period to focus on the main causes of delay; however, other studies for longer periods are recommended.

Since there is no sufficient evidence in the literature, additional studies are needed to get a consensus of what is an acceptable amount of time before tagging a discharge as delayed, so that there will be a target in the future for quality improvement.

The main reasons for delayed discharge in developing countries are different from those in developed countries. In our hospital, system-related factors were the most commonly reported in association with delayed discharge; however, medical factors and family-related factors were associated with longer delays. This study was conducted in one University hospital, thus further research is needed to examine discharge delays in other settings.

## Dissemination of results

The findings from this paper have not been disseminated beyond this publication.

## Authorship contribution statement

Authors contributed as follow to the conception or design of the work; the acquisition, analysis, or interpretation of data for the work; and drafting the work or revising it critically for important intellectual content: IE contributed 50%; WM 15%; ME, JH and ME 10%; and HE 5%. All authors approved the version to be published and agreed to be accountable for all aspects of the work.

## Declaration of competing interest

The authors declare no conflicts of interest.
